# P-2003. Comparative Performance of a Deep Learning-Based Tool (“StrepApp”) with Clinical Prediction Scores for Diagnosis of GAS Pharyngitis in Pediatric Patients

**DOI:** 10.1093/ofid/ofaf695.2167

**Published:** 2026-01-11

**Authors:** Rana F Hamdy, Youness Arjoune, Trong Nguyen, Jeffrey S Dome, Emily Ansusinha, Amir Khazraei, David Mathison, Maya Dawson, Patrick Dolan, Raj Shekhar

**Affiliations:** Childrens National Hospital, Washington, DC; Children's National Research Institute, Washington, District of Columbia; Children's National Research Institute, Washington, District of Columbia; Children’s National Hospital, Washington, District of Columbia; Children's National Hospital, Washington, District of Columbia; Auscultech, Washington, District of Columbia; PM Pediatric Care, Bethesda, Maryland; Children’s National Hospital, Washington, District of Columbia; PM Pediatrics, Mount Prospect, Illinois; Children's National Hospital, Washington, District of Columbia

## Abstract

**Background:**

Clinical signs and symptoms alone cannot reliably identify pharyngitis caused by Group A Streptococcus (GAS). Clinical prediction rules have been developed as tools for ruling out GAS. Our team has developed StrepApp -- a deep learning-based diagnostic tool that integrates pharyngeal imaging and clinical data to rule out GAS pharyngitis. The purpose of this study is to compare the performance of three tools: existing clinical prediction rules, a model using clinical data alone, and StrepApp.

**Table 1:**
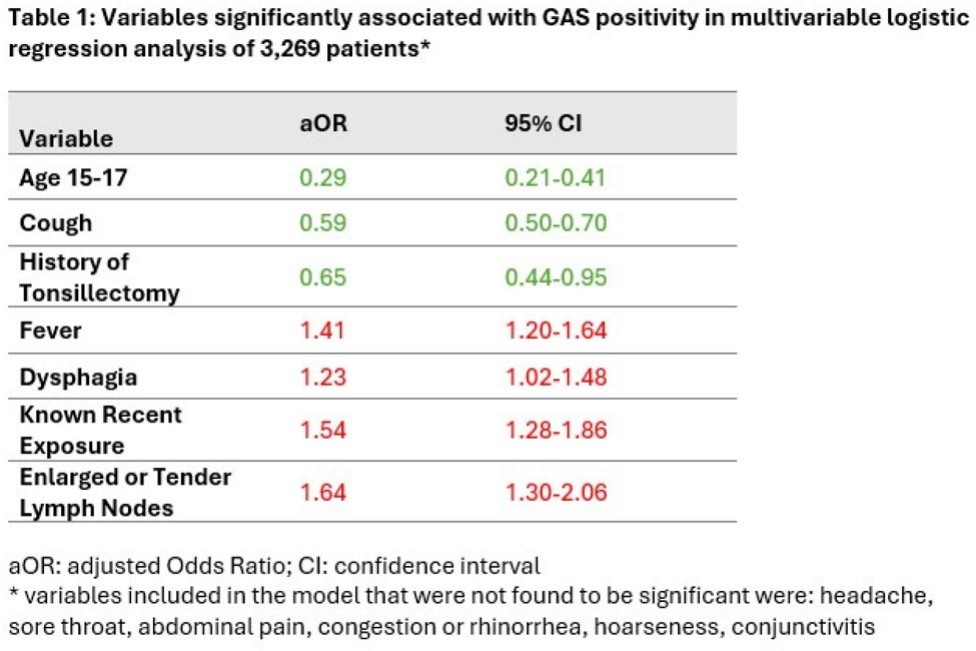
Variables significantly associated with GAS positivity in multivariable logistic regression analysis of 3,269 patients

**Figure:**
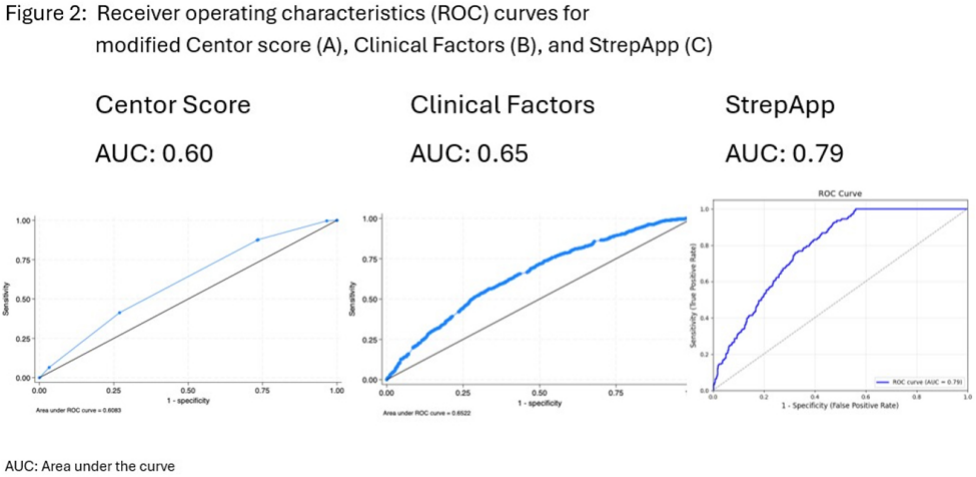
Receiver Operating Characteristic (ROC) Curves for (a) Modified Centor Score, (B) Clinical Factors, and (C) StrepApp

**Methods:**

Patients 3-17 years undergoing GAS testing were prospectively enrolled from eight U.S. pediatric urgent care or emergency department sites. Patients were excluded if they had received antibiotics within 48 hours. A custom mobile app captured throat images and clinical data. A multivariable logistic regression model was built to determine the association between each clinical variable with GAS positivity. Images were also analyzed using a multimodal deep learning method, integrating clinical data and images. Patients with upper respiratory symptoms and a positive GAS test were excluded from the image analysis. We computed the area under the receiving operator characteristics (ROC) curve for: 1) the modified Centor score, 2) clinical signs and symptoms associated with GAS positivity in the regression model (Table 1), and 3) StrepApp.

**Results:**

The database included 3,269 pediatric patients, 41.8% positive for GAS. For deep learning, images from 2,726 patients were used (1916 for training, 301 for validation and 509 for test). The variables associated with GAS positivity in the regression analysis included fever, sore throat, absence of cough, known recent exposure, and enlarged or tender cervical lymph nodes, while age >15 years and history of tonsillectomy were negatively associated with GAS (Table 1). The area under the curve (AUC) for Centor score was 0.60, for the model based on factors listed in Table 1 was 0.65, and for StrepApp was 0.79 (Figure 2).

**Conclusion:**

StrepApp demonstrated a higher AUC than clinical prediction tools. StrepApp has the potential to transform pediatric healthcare by enabling real-time artificial intelligence-powered GAS diagnosis, reducing dependency on in-person microbiologic tests that may be inaccessible in telemedicine settings.

**Disclosures:**

Rana F. Hamdy, MD, MPH, MSCE, FPIDS, No company, but co-inventor on a pending patent for StrepApp: co-inventor on a pending patent for StrepApp Jeffrey S. Dome, MD, PhD, Pixcare: We filed a provisional patent for the StrepApp technology--phone app to distinguish strep from viral pharyngitis Raj Shekhar, PhD, AusculTech Dx: Board Member|AusculTech Dx: Grant/Research Support|AusculTech Dx: Co-inventor of a pending patent application|AusculTech Dx: Ownership Interest

